# Clinical characteristics of sitosterolemic children with xanthomas as the first manifestation

**DOI:** 10.1186/s12944-022-01710-1

**Published:** 2022-10-13

**Authors:** Jun Zhang, Qiu-li Chen, Song Guo, Yan-hong Li, Chuan Li, Ru-jiang Zheng, Xue-qun Luo, Hua-mei Ma

**Affiliations:** 1 Department of Pediatrics, the First Affiliated Hospital, Sun Yat-sen University, 58# Zhong Shan 2nd Road, Yue Xiu District, GuangZhou, China; 2 The Second Affiliated Hospital of GuangXi Medical University, GuangXi, China

**Keywords:** Xanthoma, Sitosterolemia, Mean platelet volume, Anemia, Familial hypercholesterolemia, Plant sterols

## Abstract

**Background:**

Sitosterolemia (STSL) is an extremely rare genetic disease. Xanthomas as the first symptom are frequently misinterpreted as familial hypercholesterolemia (FH) in children. Inappropriate treatment may deteriorate the condition of STSL.

**Objectives:**

To present the clinical and laboratory characteristics of xanthomatous children diagnosed with sitosterolemia in comparison with childhood FH with xanthomas.

**Methods:**

We summarized and compared the clinical characteristics of STSL and FH patients with xanthomas as the first manifestations and investigated the different indicators between the STSL and FH groups, as well as their diagnostic values for STSL.

**Results:**

Two tertiary pediatric endocrinology departments contributed ten STSL cases. Five of the STSL patients (50%) experienced mild anemia, whereas two (20%) had vascular complications. The xanthomas of the STSL group displayed morphologies comparable to those of the FH group. There were ten cases of homozygous FH (HoFH) with xanthomas as the predominant symptom of the control group who had no anemia. The serum cholesterol (Chol) levels of the STSL and FH groups were 12.57 (9.55 ~ 14.62) mmol/L and 17.45 (16.04 ~ 21.47) mmol/L, respectively (*p* value 0.002). The serum low-density lipoprotein cholesterol (LDL-c) levels of the STSL and FH groups were 9.26 ± 2.71 mmol/L and 14.58 ± 4.08 mmol/L, respectively (*p* value 0.003). Meanwhile, the mean platelet volume (MPV) levels of the STSL and FH groups were 11.00 (9.79 ~ 12.53) fl. and 8.95 (8.88 ~ 12.28) fl., respectively (*p* value 0.009). The anemia proportions of the STSL and FH groups were 50% and 0%, respectively (*p* value 0.033). The AUC values of Chol, LDL-c, MPV, hemoglobin (Hb) for the diagnosis of STSL were 0.910, 0.886, 0.869, 0.879, respectively. Chol ≤ 15.41 mmol/L, LDL-c ≤ 13.22 mmol/L, MPV ≥ 9.05 fl., or Hb≤120 g/L were the best thresholds for diagnosing STSL with childhood xanthomas.

**Conclusion:**

The xanthoma morphology of STSL patients resembles that of FH patients. Xanthomas as the initial symptom of a child with Chol ≤ 15.41 mmol/L, LDL-c≤13.22 mmol/L, MPV ≥ 9.05 fl., or Hb≤120 g/L, he was most likely to have STSL.

## Introduction

Sitosterolemia (STSL, OMIM #210,250, #618,666), also known as phytosterolemia, is a rare autosomal recessive inherited lipid metabolism disease that is characterized by increased intestinal absorption and decreased bile excretion, resulting in a significant increase in serum phytosterols (such as β-sitosterol, campesterol, and stigmasterol)[1, 2]. STSL was first documented in two Amish sisters in 1974 by Bhattacharyya and Connor. Increased plasma phytosterol levels and tendon xanthomas were observed[3]. Xanthomas as the first symptom are frequently misinterpreted as familial hypercholesterolemia (FH) in children[1, 4]. STSL was originally predicted to have a prevalence of less than 1/million[5], but this figure may be conservative. According to the Exome Aggregation Consortium, 1 in ~ 220 individuals has a loss-of-function mutation in either the ABCG5 or ABCG8 gene[6]. According to this information, the disease affects approximately 7000 people in China and more than 30,000 persons worldwide. However, there are currently just over 100 cases of STSL reported worldwide. Studies of patients with hypercholesterolemia showed that the diagnosis of sitosterolemia is obviously underestimated and delayed[7, 8].

These people may undergo prolonged, inappropriate high-risk treatment as a result of the delay or inaccuracy in diagnosis[9]. They may miss the opportunity to benefit from a low-cholesterol and low-phytosterol diet or ezetimibe/bile acid sequestrant therapy[10]. Particularly in terms of treatment, STSL requires strict control of cholesterol and plant sterol intake, while European guidelines of FH recommend a daily plant sterol intake of 2 g[11].

Challenges in the proper diagnosis of sitosterolemia include heterogeneity of the clinical characteristics of the disease, and the inability of routine clinical laboratory assays to distinguish phytosterols from cholesterol. At present, the best diagnostic method is gas chromatography-mass spectrometry (GC–MS) for detecting blood phytosterols, but many regions and hospitals have not yet implemented GC–MS for phytosterol detection. GC–MS for plant sterols in China has not been popularized in clinical laboratories, and a reference interval for plant sterols based on the Chinese population has not been established. Another good diagnostic tool is genetic analysis, but genetic testing is time-consuming and expensive. Nevertheless, it is a reliable method for early diagnosis in regions or countries where blood plant sterols cannot be detected.

STSL also has other major clinical manifestations, such as giant platelets with macrothrombocytopenia (reduced giant platelet count) and hemolytic anemia[12–16]. Therefore, this study intends to explore whether there are differences in cholesterol (Chol), low-density lipoprotein cholesterol (LDL-c), mean platelet volume (MPV), and hemoglobin (Hb) between children with STSL and FH with xanthomas as the primary clinical presentation, as well as to summarize other clinical features of STSL, to provide clues for early clinical diagnosis.

## Objectives and methods

### Subjects

Children with STSL with xanthomas as the first presentation from January 2016 to October 2021 were retrospectively and continuously included as a case group, with 9 cases from the Pediatric Growth and Development Center of the First Affiliated Hospital,  Sun Yat-sen University and 1 case from The Second Affiliated Hospital of GuangXi Medical University. During the same period, ten children with xanthomas as the primary manifestations who were diagnosed with FH at the Pediatric Growth and Development Center of the First Affiliated Hospital, Sun Yat-sen University were retrospectively and continuously included as the control group.

The inclusion criteria for STSL cases were as follows: (1) 0–14 years old; (2) presented with a chief complaint of xanthomas; (3) diagnosis of STSL confirmed by genetic analysis – homozygous variants of ABCG5 and ABCG8 or compound heterozygous variants[2]. The exclusion criteria for STSL cases were as follows: (1) other diseases manifested by xanthomas, such as cerebrotendinous xanthomatosis, Wolman disease, and FH; (2) other secondary hyperlipidemia with elevated LDL-c, such as hypothyroidism and nephrotic syndrome; (3) those with abundant missing data; and (4) those with the treatment of dietary restriction and/or lipid-lowering drugs.

The inclusion criteria for the FH cases were as follows: (1) 0–14 years old; (2) presented with a chief complaint of xanthoma; (3) laboratory tests suggesting LDL-c≥3.6 mmol/L; (4) diagnosis of FH confirmed by genetic testing – homozygous or heterozygous mutations of LDLR, ApoB, ApoE, or PCSK9 or homozygous or compound heterozygous mutations of LDLRAP1[11]. The exclusion criteria for the FH cases were as follows: (1) other diseases manifested by xanthomas, such as cerebrotendinous xanthomatosis, Wolman disease, and STSL; (2) other secondary hyperlipidemia with elevated LDL-c, such as hypothyroidism and nephrotic syndrome; (3) those with abundant missing data; and (4) those with the treatment of dietary restriction and/or lipid-lowering drugs.

This study was approved by the Medical Ethics Committee of the First Affiliated Hospital, Sun Yat-sen University. Written consent was obtained from parents and children.

### Methods

1. For the case data, the following information was collected and recorded: the children’s sex and ancestry; the parental height and lipid values; the age of onset and diagnosis, medical history, clinical course, whether xanthoma biopsy was performed, and medication use at the first visit to the First Affiliated Hospital, Sun Yat-sen University or The Second Affiliated Hospital of GuangXi Medical University; the location, numbers, maximum diameter (cm) of xanthomas; height, and weight.
2. The determination of routine blood tests, blood lipids, and other indicators included the following: inclusion of Hb, mean corpuscular volume (MCV), platelets (PLT), and MPV in routine blood tests (SYSMEX, XN9000, Japan); Chol, triglycerides, high-density lipoprotein cholesterol (HDL-c), and LDL-c (Beckman, AU5800, USA); assessment of cardiovascular complications, including coronary, carotid, and aortic ultrasound (EPIC7C, Philips, The Netherlands); and cranial MRI angiography (Verio, Syngo MR B19, Siemens Healthcare, Germany).
3. Anemia was identified when the Hb concentration fell below a defined threshold. Children 1–3 months of age (MOA): Hb < 90 g/L; 4–5 MOA: Hb < 100 g/L; 6–59 MOA: Hb < 110 g/L; 5–11 years of age (YOA): Hb < 115 g/L; 12–14 YOA: Hb < 120 g/L.
4. Next-generation sequencing.

With the informed consent of the child and parents, genomic DNA was extracted from peripheral blood by a standard procedure. The extracted DNA was fragmented with DNAase and purified by the magnetic particle method. The extracted DNA was then amplified by PCR and ligated to the ligation sequence. After capturing and purifying two times with xGen Exome Research Panel V1.0 (IDT, USA), followed by amplification by PCR and purification, the final DNA libraries were obtained. The exons and exon-intron junction regions of the target genes (LDLR, PCSK9, APOB, LDLRAP1, LIPA, CH25H, SREBF1, ABCG5, ABCG8, SCAP, STAP1, MYLIP, APOE, NPC1L1, LPA, LPL, APOA5, GPIHBP1, GPD1, LMF1, and CREB3L3) were sequenced and analyzed on a NovaSeq 6000 sequencer (Illumina Inc, USA). All data were compared to the reference sequence (UCSC hg19) using the BWA algorithm. The default settings of the instrument were used, and the data were annotated using literature-reported methods. The functions, variants, and inheritance patterns of each gene were analyzed according to the ACMG guidelines combined with the clinical data and the prediction results of bioinformatics software (PolyPhen2, LRT, Mutation Taster, etc.). Then, candidate variants were obtained. PCR primers were designed to amplify the fragments of the candidate variants. Sanger sequencing was performed to validate the variants, and the corresponding variant loci of the parents were analyzed and validated. The high-throughput sequencing tests were performed by third-party inspection agencies. Most of them were done by KindMed.

### Statistical analysis

All analyses were conducted using the SPSS 26.0 statistical package for Mac (IBM SPSS Inc., Chicago, IL, USA). The distribution of data was assessed by using the Kolmogorov–Smirnov test. Values with a normal distribution are expressed as the mean ± SD, and values with a nonnormal distribution are expressed as the median (interquartile range, IQR). Categorical data are expressed as frequencies and percentages. Differences between the two groups were compared with Student’s t test and the Mann–Whitney U test for normally distributed variables and nonnormally distributed variables, respectively. Differences in categorical data were analyzed using the chi-square test (Fisher’s exact method). Paired data (Chol and LDL-c with parental Chol and LDL-c in the STSL group; height SDS with target height SDS) were analyzed using paired-sample t tests or Wilcoxon’s signed-rank test. A receiver operating characteristic curve (ROC) was conducted to compare observer performance for Chol, LDL-c, MPV, and the presence of anemia. Optimal cutoff points for the indicators were determined with Youden’s J statistic using the following equation: Jmax. = Sensitivity + Specificity – 1. The index values corresponding to the maximum value of Youden’s J statistic were recognized as optimal cutoff points for these indices. A value of *p* < 0.05 was considered statistically significant.

## Results

### Overall demographic and clinical characteristics

The number of eligible cases was ten for the STSL group, ten for the FH group, and zero for exclusion due to abundant missing data.

**Fig. 1:**
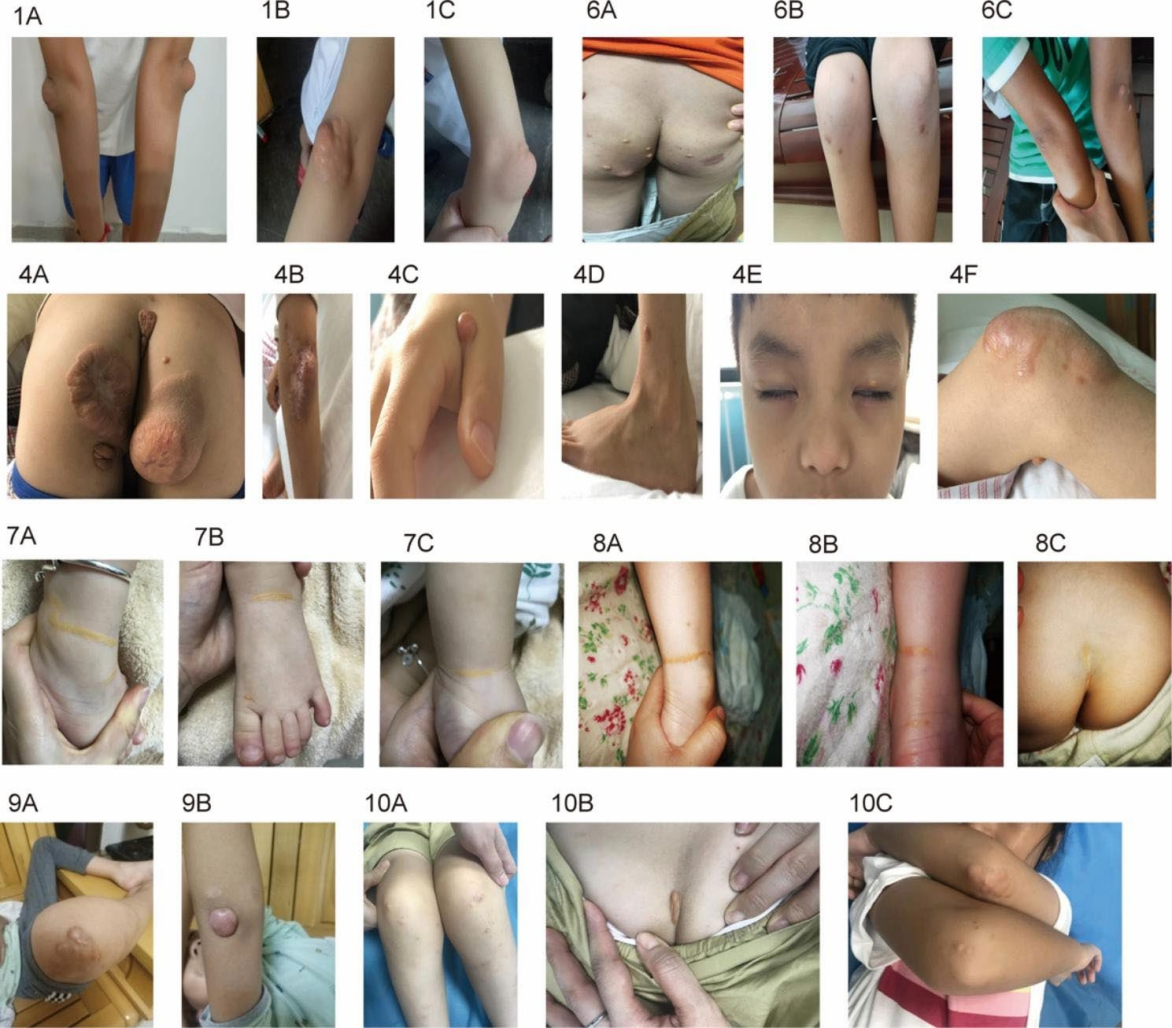
Xanthomas of STSL patients. The numbers are the case numbers in Table 1

STSL patients with xanthomas as the first manifestation were included as the case group (Table 1), which included five males and five females from nine pedigrees. Case 10 was from Guangxi Province, while the rest were from Guangdong Province. All patients were Han Chinese, with nonconsanguineous parents. There was no previous history of hemorrhage or hemolytic disease and no family history of xanthomas. None of them exhibited arthralgia. Xanthomas are a result of cutaneous lipid deposition and present with varying morphologies. Most of the xanthomas were tuberous, and some were fused (Fig. 1 and 1-B, 9-A). Some large nodules presented lobulated shapes (Figs. 1 and 4-A). Some appeared in the infancy and toddler periods with linear and intertriginous xanthomas (Figs. 1 and 7 A-C, 8 A-C), and some showed a patchy shape (Figs. 1 and 7-A). The most common distribution sites were the metacarpophalangeal, wrist, elbow, knee, ankle, buttock groove, buttock, eyelid, and Achilles tendon at the extensor areas (Fig. 1). Five cases (50%) had mild anemia, with the exclusion of iron deficiency anemia and thalassemia, whereas stomatocytes were not found in peripheral blood smear of the patients with anemia. There were no abnormalities in routine urine tests, liver and kidney function, thyroid function, or adrenal function. All patients had Doppler ultrasonography of the liver and spleen, and they revealed no hepatosplenomegaly or cholelithiasis. Six cases (6/10, 60%) in the STSL group had a xanthoma biopsy, which exhibited numerous foam cells and indicated xanthomas. They all performed a vascular assessment and the images of two cases (Case 4 and Case 6) showed vascular stenosis without clinical manifestations. Specifically, for case 4, stenosis was present at the beginning segment of the right subclavian artery (< 50%) and bilateral jugular stenosis (< 50%). Case 4 was reported in Frontier in Pediatrics in 2021[17]. The imaging of Case 6 suggests severe stenosis at the beginning of the left anterior cerebral artery. They are currently being followed for more than 2 and 3 years, respectively. Thus far, they have all been in good health without experiencing any ischemia symptoms.

**Table 1:** Clinical features of the STSL group

Case	Age at diagnosis (years)	Height (cm)	Features of xanthomas	Chol (mmol/L)	LDL-c (mmol/L)	Hb (g/L)	PLT (×10^9^/L)	MPV (fl.)	Gene sequences
1	11.04	136.0	①②③	13.23	7.39	112	113	11.40	ABCG5, Exon 3, c.335dupA, p. (Val113fs), He, from Father. ABCG5, Exon 6, c.751C>T, p. (Gln251*), He, from Mother.
2	0.36	66.2	①②	9.40	6.89	107	336	-	ABCG5, Exon 10, c.1337G>A, p. (Arg446Gln), He, from Mother. ABCG5, Exon 3, c.335dupA, p. (Val113fs), He, from Father.
3	8.83	124.0	①②	9.60	6.70	124	190	12.90	ABCG5, Exon 10, c.1336 C>T, p. (Arg446*), He, from Mother. ABCG5, Exon 9, c.1166G>A, p. (Arg389His), He, from Father.
4	8.00	114.6	①②⑤	14.49	12.93	108	253	13.60	ABCG5, Intron 7, c.904 + 1G>A, p.?, He, from Mother. ABCG5, Intron 9, c.1324 + 1delG, p.?, He, from Father.
5 (Sister of case 4)	6.16	112.0	①②	7.80	5.46	113	346	11.00	ABCG5, Intron 7, c.904 + 1G>A, p.?, He, from Mother. ABCG5, Intron 9, c.1324 + 1delG, p.?, He, from Father.
6	11.88	139.6	①②	11.90	8.60	104	252	11.00	ABCG5, Exon 6, c.751 C>T, p. (Gln251*), He, from Mother. ABCG5, Exon 11, c.1528 C>G, p. (His510Asp), He, from Father.
7	1.24	75.0	①②④⑥	15.02	12.24	107	418	9.50	ABCG5, Exon 10, c1336C>T, p. (Arg446*), He, from Father. ABCG5, Exon 9, c.1166G>A, p. (Arg389His), He, from Mother.
8	3.04	94.0	①④	14.39	12.74	131	330	9.10	ABCG8, Exon 11, c.1720G>A, p. (Gly574Arg), He, from mother. ABCG8, Exon 12, c.1877G>T, p. (Gly626Val), He, from Father.
9	7.68	118.0	①②	15.00	10.35	116	293	10.64	ABCG5, Intron 7, c.904 + 1G>A, Ho, from mother and father, respectively
10	10.73	130.0	①②③	11.30	9.25	123	272	-	ABCG5, Exon 6, c.751 C>A, p. (Gln251*), He, from father. ABCG5, Exon 3, c.335dupA, p. (Val113fs), He, from mother.
Reference range				2.90–5.17	2.07–3.36		100–300	9.00–13.00	

MPV, mean platelet volume; ①, multiple; ②, tuberous; ③, fused; ④, intertriginous; ⑤, lobulated; ⑥, patchy; He, heterozygous; Ho, homozygous; -, Missing data

HoFH cases with xanthomas as the first presentation were included as controls (Table 2), which included six girls and four boys from eight Chinese pedigrees. The morphology of the xanthomas in the FH group also presented as multiple nodules, partially fused or linear, similar to that of the STSL group. There were no cases of anemia. Four patients (4/10, 40%) had a histological examination of the xanthomas in the FH group, exhibiting numerous foam cells that indicated xanthomas.

**Table 2:** Clinical characteristics of the FH group

Case	Age at diagnosis (years)	Height (cm)	Features of xanthomas	Chol (mmol/L)	LDL-c (mmol/L)	Hb (g/L)	PLT (×10^9^/L)	MPV (fl.)	Gene sequences
1	1.32	77.0	①②④	29.4	21.02	126	316	9.00	Compound heterozygous variants of LDLR, c.1241T>G, p. (Leu414Arg), from mother, c.1327T>C, p. (Trp443Arg), from father.
2 (Twin of case 1)	1.32	77.2	①④	27.3	19.7	124	354	8.90	Compound heterozygous variants of LDLR, c.1241T>G, p. (Leu414Arg), from mother, c.1327T>C, p. (Trp443Arg), from father.
3	10.11	134.0	①②③	18.7	15.95	132	284	8.60	Compound heterozygous variants of LDLR, c.1241T>G, p. (leu414Arg), from father, c.1073G>T, p. (Cys358Phe), from mother.
4 (Younger sister of case 3)	3.12	98.0	①②	17.4	14.25	112	289	9.10	Compound heterozygous variants of LDLR, c.1241T>G, p. (leu414Arg), from father, c.1073G>T, p. (Cys358Phe), from mother.
5	5.47	104.3	①②	16.12	14.14	116	372	8.80	Compound heterozygous variants of LDLR, c.681 C>G, p. (Asp227Glu), from mother, c.1241T>G, p. (Leu414Arg), from father.
6	12.24	152.2	①②	8.2	6	125	216	10.10	Compound heterozygous variants of LDLR, c.226>G, p. (Gly76Arg), from mother, c.1474G>A, p. (Asp492Asn), from father.
7	7.29	120.1	①②	19.53	13.5	132	243	10.80	Homozygous variants of LDLR, c.2389G>A, p. (Val97Met), from mother and father, respectively.
8	8.47	127.4	①②	17.5	14.34	139	239	8.90	Compound heterozygous variants of LDLR, c.888 C>A, p. (Cys96*), from father, c.1198T>A, p. (Tyr 400Asn), from mother.
9	13.35	162.0	①②	15.8	14.56	148	260	11.30	Compound heterozygous variants of LDLR, c.482T>C, from father, c.827G>A, from mother.
10	7.44	113.0	①②	16.6	12.36	130	332	8.90	Compound heterozygous variants of LDLR, c.1241T>G, p. (Leu414Arg), c.681 C>G, p.(Asp227Glu)
Reference range				2.90–5.17	2.07–3.36		100–300	9.00–13.00	Compound heterozygous variants of LDLR, c.1241T>G, p. (Leu414Arg), from father, c.681 C>G, p. (Asp227Glu), from mother.

MPV, mean platelet volume; ①, multiple; ②, tuberous; ③, fused; ④, intertriginous

The Chol values (12.57 (9.55 ~ 14.62) mmol/L) of STSL group patients were significantly higher than those of the corresponding fathers (5.85 (5.20 ~ 7.08) mmol/L) and mothers (5.22 ± 0.50 mmol/L) (the *p* values were 0.012 and 0.018, respectively). The STSL patients’ LDL-c values (9.26 ± 2.71 mmol/L) were significantly higher than those of their fathers (3.82 (3.51 ~ 4.76) mmol/L) and mothers (2.99 ± 0.51 mmol/L) (the *p* values were 0.012 and 0.018, respectively). Although STSL is an autosomal recessive disorder, the mean values of the parental Chol were also elevated in the STSL group (Table 3). There was no thrombocytopenia in either the STSL or FH group (Tables 1 and 2), while four patients in the STSL group had elevated platelet counts.

**Table 3:** Comparison between the STSL group and FH group

Indicators	STSL Group	FH Group	*P* values
Male, n (%)	5 (50%)	4 (40%)	1.00
Age at diagnosis(years), mean ± SD	6.90 ± 4.12	7.01 ± 4.24	0.951
The interval between onset and diagnosis (years), median (IQR)	1.50 (0.96 ~ 2.56)	1.00 (0.73 ~ 3.75)	0.675
SDS of height, mean ± SD	-0.95 ± 1.21	-0.58 ± 0.87	0.440
BMI (kg/m^2^), mean ± SD	14.84 ± 2.25	15.39 ± 1.70	0.543
Chol (mmol/L), median (IQR) Reference range: 2.90–5.17 mmol/L	12.57 (9.55 ~ 14.62)	17.45 (16.04 ~ 21.47)	0.002
LDL-c (mmol/L), mean ± SD Reference range: 2.07–3.36 mmol/L	9.26 ± 2.71	14.58 ± 4.08	0.003
Hb (g/L), mean ± SD	114.50 ± 8.89	128.40 ± 10.46	0.005
Anemia, n (%)	5 (50%)	0 (0%)	0.033
MCV (fl.), median (IQR)	81.34 (69.35 ~ 83.40)	81.25 (74.50 ~ 85.13)	0.762
PLT (×10^9^/L), mean ± SD	280.34 ± 86.13	290.50 ± 52.15	0.753
MPV (fl.), median (IQR)	11.00 (9.79 ~ 12.53)	8.95 (8.88 ~ 12.28)	0.009
Paternal Chol (mmol/L), median (IQR) Reference range: 2.90–5.17 mmol/L	5.85 (5.20 ~ 7.08) (n = 8)	8.18 (6.95 ~ 8.40) (n = 10)	0.008
Paternal LDL-c (mmol/L), median (IQR) Reference range: 2.07–3.36 mmol/L	3.82 (3.51 ~ 4.76) (n = 8)	5.64 (4.93 ~ 6.13) (n = 10)	0.021
Maternal Chol (mmol/L), mean ± SD Reference range: 2.90–5.17 mmol/L	5.22 ± 0.50 (n = 8)	6.97 ± 1.28 (n = 10)	0.004
Maternal LDL-c (mmol/L), mean ± SD Reference range: 2.07–3.36 mmol/L	2.99 ± 0.51 (n = 8)	4.79 ± 1.01 (n = 10)	0.001

SDS of height, standard deviation score of height; MCV, mean corpuscular volume; MPV, mean platelet volume

### Gene sequencing results

In the STSL group, eight cases were compound heterozygous variants, and one case was a homozygous variant of the ABCG5 gene. One case was a compound heterozygous variant of the ABCG8 gene (Table 1) (All of the original data have been uploaded to the SRA database with BioProject ID: PRJNA838408). Ten pathogenic variants were identified, and all were reported. The highest frequency of variants in this group of patients was IVS7 + 1G > A, followed by Gln251* and Val113fs.

In the FH group, the gene sequencing results showed one case (case 7) of homozygosity and 9 cases of compound heterozygous variants of LDLR. Therefore, all subjects in the control group with xanthomas as the first manifestation were HoFH.

### Clinical data of the STSL and FH groups

One case in the STSL group (Table 1, case 4) and one in the FH group (Table 2, case 10) met the diagnostic criteria for short stature. However, the standard deviation score (SDS) of height in all twenty cases was − 0.84 ± 1.02, which did not differ from the target height SDS (-0.38 ± 0.60) with a *p* value of 0.063. There was also no difference in the SDS of height between the STSL (-0.95 ± 1.21) and FH (-0.58 ± 0.87) groups (*p* value 0.44) (Table 3). There were no differences in body mass index (BMI; weight (kg)/height (m)^2^), clinical course, PLT, or MCV between the two groups (Table 3). Chol and LDL-c levels in the STSL group were considerably lower than in the FH group (Table 3). The STSL group’s parental Chol and LDL-c levels were significantly lower than those of the FH group (Table 3). Additionally, MPV and anemia ratio of STSL group were much higher than those of the FH group (Table 3). Meanwhile, Hb was considerably lower in the STSL group compared to the FH group (Table 3).

### Receiver operating curve (ROC) analysis

**Fig. 2:**
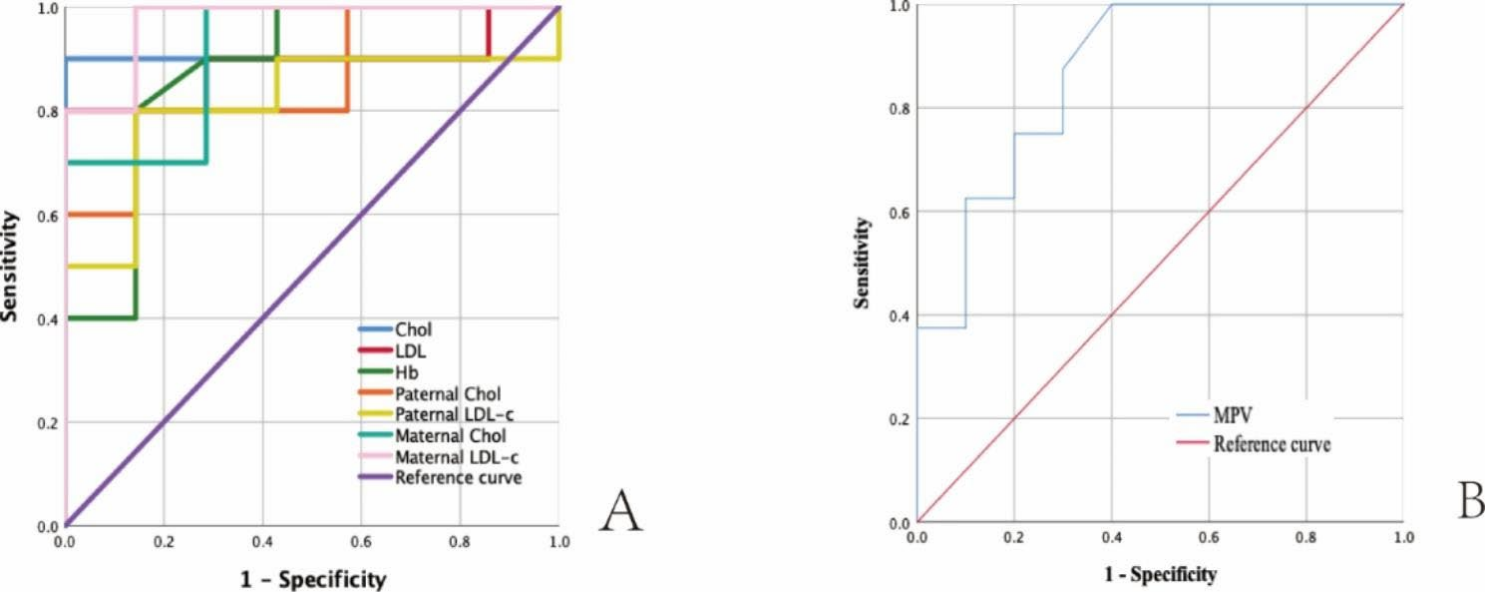
ROCs of indicators for the diagnosis of STSL

To better distinguish whether a child presenting with xanthomas as the first manifestation was STSL or FH, ROCs were drawn for the diagnosis of STSL using Chol, LDL-c, MPV, Hb, parental Chol, parental LDL-c (Fig. 2). All indicators had good diagnostic values for the area under the curve (AUC) (Table 4; Fig. 2). Their Youden’s J statistic values were calculated separately. The diagnostic cutoff value of each indicator was taken at the maximum of Youden’s J statistic. Chol≤15.41 mmol/L, LDL-c≤13.22 mmol/L, MPV≥9.05 fl., or Hb≤120 g/L were the best cutoff values for the diagnosis of STSL in the childhood patient with xanthomas.

**Table 4:** AUC values and cutoff values of indicators for the diagnosis of STSL

	AUC values (95% CI)	*p* value of AUC	Maximum of Youden’s J statistic	Cutoff values for the diagnosis of STSL		
				Cutoff value	Sensitivity	Specificity
Chol	0.910 (0.751-1.000)	0.005	0.9	≤ 15.41 mmol/L	0.9	1.0
LDL-c	0.886 (0.712-1.000)	0.008	0.8	≤ 13.22 mmol/L	0.8	1.0
MPV	0.869 (0.704-1.000)	0.009	0.6	≥ 9.05 fl.	1.0	0.6
Hb	0.879 (0.700-1.000)	0.010	0.8	≤ 120 g/L	0.8	0.9
Paternal Chol	0.857(0.677-1.000)	0.015	0.7	≤ 7.25 mmol/L	0.8	0.9
Paternal LDL-c	0.814(0.597-1.000)	0.032	0.7	≤ 5.02 mmol/L	0.8	0.9
Maternal Chol	0.914(0.776-1.000)	0.005	0.7	≤ 5.25 mmol/L	1.0	0.7
Maternal LDL-c	0.971(0.901-1.000)	0.001	0.9	≤ 3.43 mmol/L	1.0	0.9

## Discussion

The clinical features of STSL overlapped with HoFH[1]. STSL is frequently misdiagnosed as FH in the childhood patient with xanthomas[1, 4]. Therefore, identifying the differences between STSL and FH based on the clinical traits will aid in guiding the diagnosis and help decrease misdiagnosis. This article revealed that lower serum Chol and LDL-c levels, lower Hb, especially when MPV ≥9.05 fl., were highly suggestive of STSL when the childhood patient presented primarily with xanthomas.

Xanthomas in STSL are morphologically diverse and usually found on the extensor surfaces of extremity joints, eyelids, and buttocks. Minor trauma plays a vital role in xanthoma development, and therefore, they appear on extensor surfaces in most patients[18]. The morphology of xanthomas in the HoFH group was similar to that of the STSL group, and biopsies of the xanthomas could not distinguish between STSL and HoFH. Only <15% of heterozygous FH (HeFH) patients aged 20–79 years developed xanthomas in the Spanish FH cohort study, so HeFH often has an insidious onset and may be missed[19]. However, in untreated HeFH individuals, the incidence of xanthomas increases with age[19, 20]. Therefore, all of the controls being HoFH may be strongly related to the inclusion of children aged 0–14 years. As the 2015 American Heart Association stated [21], HoFH patients usually show xanthomas before 10 years of age, whereas HeFH patients generally do not manifest xanthomas until adulthood.

Vascular stenosis was found in two cases (20%, 2/10) of STSL in this study, representing the early-onset cardiovascular events of STSL. Xia[16] and Tada[14] reported that 19% and 25% of STSL patients exhibited atherosclerosis, respectively. Phytosterols can penetrate the arterial wall, stimulate foam cell formation, produce proinflammatory cytokines, attract more monocytes and accelerate atherosclerosis[22]. Additionally, the accumulation of β-sitosterol leads to the death of macrophages, which may accelerate the necrosis of atherosclerotic plaques[22]. In addition, phytosterols are highly sensitive to oxidative processes compared to cholesterol, and phytosterol oxidation products can be proinflammatory and proatherogenic[23]. Nevertheless, the specific atherogenic potential of phytosterols is not known to date.

The gene sequencing results suggested that nine out of ten cases were homozygous or compound heterozygous variants of the ABCG5 gene, and only one case was a compound heterozygous variant of the ABCG8 gene. This is consistent with previous reports that Caucasians often carry ABCG8 variants, while Chinese, Japanese and Indian patients tend to have ABCG5 variants [14–16, 24, 25]. In addition, the pathogenic variant with the highest frequency in this study was IVS7 + 1G$>$A. Su et al. summarized 28 Chinese STSL cases reported in the literature and revealed that more than half were Arg446* variants of the ABCG5 gene[26]. Xia and her colleagues reported that c.694+5G>C (p. Tyr209Asnfs∗43) of the ABCG5 gene was the most common variant in a cohort study of sixty Chinese STSL cases [16]. Other studies have also suggested that the most common variants of the ABCG5 gene are Arg389His and Arg419His[27, 28]. Therefore, the common pathogenic variants of the ABCG5 gene need to be studied in larger samples. Furthermore, Bastida et al. summarized the pathogenic variants with macrothrombocytopenia, such as Arg446*, Arg446Gln, IVS7+1G>A, and IVS9+1delG [29]. However, the phenotype of these variants in this study corresponded to average PLT counts or even slightly elevated counts. The same genotype may have different clinical phenotypes. This may be due to other coexisting genes involved in phytosterol metabolism and/or environmental impacts on hematopoietic cells.

Although STSL is a recessive inherited disorder, this study observed elevated Chol levels in the parents of the patients with heterozygous variants of ABCG5/8. Some papers have also reported higher cholesterol and phytosterol levels in ABCG5/8 heterozygous variants [30–32]. Furthermore, there is a case report of a 48-year-old woman misdiagnosed with HeFH due to elevated LDL-c since the age of 20. Treatment with statins was ineffective, but treatment with ezetimibe was excellent. Eventually, it was confirmed that she had an ABCG5 heterozygous variant [33]. These findings suggest that the ABCG5/8 heterozygous variant may also affect cholesterol and phytosterol metabolism.

This study found that the HoFH group had higher Chol and LDL-c levels than the STSL group. Additionally, Chol and LDL-c levels in the STSL group were elevated and significantly higher than the corresponding paternal and maternal levels (Table 3). This is consistent with Wang et al., who reported that in 13 STSL patients, eight had elevated LDL-c, and ten had elevated Chol levels[34]. Furthermore, Xia also revealed that the elevation of Chol and LDL-c was observed in 96% of Chinese STSL individuals. Therefore, receiver operating curves of Chol and LDL-c were used to explore the best cutoff values to distinguish STSL from HoFH. The AUC values of Chol and LDL-c for the diagnosis of STSL were 0.910 and 0.886, respectively, both of which had good diagnostic values. STSL was more likely when Chol ≤ 15.41 mmol/L or LDL-c ≤ 13.22 mmol/L, which could help to better diagnose children with xanthomas as the first complaint.

Macrothrombocytopenia or hemolytic anemia with STSL has been reported in the literature[13, 14, 16, 34, 35]. Of the ten patients with STSL in this study, 50% had mild anemia. Iron deficiency anemia and thalassemia, which are common in southern China, were excluded. Anemia might be related to the primary disease. In addition, the MPV was greater in the STSL group than in the FH group. This result suggested that although thrombocytopenia did not occur in the STSL patients, the elevation of phytosterols in the blood may have had a morphological effect on platelets as well. Studies in ABCG5- and ABCG8-deficient mice have shown that the accumulation of phytosterols in the circulation promotes cell membrane stiffness and makes them prone to rupture, leading to morphological and functional abnormalities[36]. Therefore, this study explored the value of the MPV and Hb in the diagnosis of STSL. The AUC values of the MPV and Hb for diagnosing STSL were 0.869 and 0.879, respectively. An MPV ≥ 9.05 fl. or Hb ≤ 120 g/L may well distinguish STSL from FH with childhood xanthomas.

There are several limitations of this study. First, this study was a retrospective case-control study with some missing data. Second, our laboratory is not yet able to perform phytosterol testing with GC–MS. Finally, the sample size of this study was not large enough. Future prospective clinical studies with larger samples are needed to further reveal the diagnostic value of Chol, LDL-c, Hb, and especially the MPV in STSL.

To the best of our knowledge, this is the first clinical research to explore the differences between STSL and FH in pediatric patients with xanthomas as the initial presentation, and to propose indicators and their cutoffs for the diagnosis of STSL. Our research might offer some clinical hints for timely diagnosis of STSL, decreasing the likelihood of misdiagnosis.

## Conclusion

STSL and FH have overlapping clinical presentations and are difficult to distinguish by conventional laboratory tests. This study revealed that a child with xanthomas as the initial symptom, along with Chol ≤ 15.41 mmol/L, LDL-c ≤ 13.22 mmol/L, MPV ≥ 9.05 fl., or Hb ≤ 120 g/L could be clinically diagnosed as STSL. It may give a more straightforward and accessible way for early diagnosis of STSL. Especially in less developed countries or regions where blood phytosterol testing by GC–MS is not available or during the gap period while waiting for gene sequencing results, the initial diagnosis can be obtained. The patient may then be prescribed proper diet management.

## Data Availability

The datasets used and/or analyzed during the current study are available from the corresponding author on reasonable request.

## List of abbreviations

FH: familial hypercholesterolemia
STSL: sitosterolemia
Chol: cholesterol
LDL-c: low-density lipoprotein cholesterol
MPV: mean platelet volume
ABCG5: adenosine triphosphate-binding cassette subfamily G member 5
ABCG8: adenosine triphosphate-binding cassette subfamily G members 8
GC–MS: gas chromatography-mass spectrometry
Hb: hemoglobin
MCV: mean corpuscular volume
PLT: platelets
HDL-c: high-density lipoprotein DL cholesterol
IQR: interquartile range
ROC: receiver operating characteristic curve
AUC: areas under the curve
HeFH: heterozygous FH
HoFH: homozygous FH
